# Modeling inherited retinal diseases using human induced pluripotent stem cell derived photoreceptor cells and retinal pigment epithelial cells

**DOI:** 10.3389/fmed.2024.1328474

**Published:** 2024-07-01

**Authors:** Ivan Seah, Debbie Goh, Animesh Banerjee, Xinyi Su

**Affiliations:** ^1^Translational Retinal Research Laboratory, Institute of Molecular and Cell Biology (IMCB), Agency for Science, Technology and Research (A*STAR), Singapore, Singapore; ^2^Department of Ophthalmology, Yong Loo Lin School of Medicine, National University of Singapore, Singapore, Singapore; ^3^Bloomberg School of Public Health, Johns Hopkins University, Baltimore, MD, United States; ^4^Department of Ophthalmology, National University Hospital (NUH), Singapore, Singapore; ^5^Singapore Eye Research Institute (SERI), Singapore, Singapore

**Keywords:** inherited retinal diseases, induced pluripotent stem cell, organoids, photoreceptor cells, retinal pigment epithelium

## Abstract

Since the discovery of induced pluripotent stem cell (iPSC) technology, there have been many attempts to create cellular models of inherited retinal diseases (IRDs) for investigation of pathogenic processes to facilitate target discovery and validation activities. Consistency remains key in determining the utility of these findings. Despite the importance of consistency, quality control metrics are still not widely used. In this review, a toolkit for harnessing iPSC technology to generate photoreceptor, retinal pigment epithelial cell, and organoid disease models is provided. Considerations while developing iPSC-derived IRD models such as iPSC origin, reprogramming methods, quality control metrics, control strategies, and differentiation protocols are discussed. Various iPSC IRD models are dissected and the scientific hurdles of iPSC-based disease modeling are discussed to provide an overview of current methods and future directions in this field.

## 1 Introduction

Inherited retinal diseases (IRDs) are a heterogenous group of monogenic retinal disorders characterized by mutations in various retinal cell types. Over 300 genes are implicated in the pathogenesis of approximately 30 IRD clinical entities such as retinitis pigmentosa (RP), Stargardt disease (SD), and Leber congenital amaurosis (LCA). Despite being individually rare, IRDs represent the largest group of untreatable diseases in ophthalmology (1). They can incur significant societal costs as many result in progressive visual loss. The prevalence varies between different IRDs, ranging from 1 in 10,000 for more common diseases such as SD to 1 in 100,00 for conditions such as choroideremia (2, 3).

The societal burden of IRDs arise from multiple aspects including the losses of individual productivity, costs associated with provision of healthcare and deadweight losses to society. Deadweight losses include government expenditure on services and facilities for the visually handicapped as well as the loss in income taxation revenue due to non-participation of patients in the workforce. In a study of the cost-of-illness of 10 most common IRDs in the United Kingdom, it was estimated that IRDs harbor a societal cost of up to £523.3 million in 2019 (4). Despite the rare disease label, these costs warrant the urgent research and development efforts into effective therapies for IRDs (4).

Despite the impact, only one Food and Drug Administration (FDA) approved therapeutic, voretigene neparvovec (Luxturna™, Novartis), is available. This is an adeno-associated viral-based gene replacement therapy for biallelic *RPE-65* mutation-associated IRDs specifically, which only represent 0.8%–1.5% of all IRDs (5). Although efforts are currently underway to develop treatments for other IRDs, a significant hurdle for success is the lack of biologically relevant disease models for evaluating the effects of therapeutics.

Among the various disease modeling approaches, using patient-derived tissue is extremely promising as it can closely capture the pathogenic mechanisms. Such models were only possible in the early 2000s due to the invention of the Nobel-prize winning induced pluripotent stem cells (iPSC) and clustered regularly interspaced short palindromic repeats (CRISPR)-based technologies (6, 7). With iPSC technology, cellular material could be derived from patients with diseases for probing of its pathological mechanisms. Meanwhile CRISPR technology enabled generation of isogenic control lines by editing the identified mutations back to its wild-type, allowing the study of genotype-phenotype correlation at a cellular level (8).

Induced pluripotent stem cell technology was first demonstrated by Nobel Prize winner, Yamanaka et al., where four transcription factors Oct 3/4, Sox2, c-Myc, and Klf4 were introduced to induce human fibroblasts back to a pluripotent state (7, 9). This had two major scientific applications: (1) an infinite source of stem cells for regenerative medicine applications and (2) patient-derived cellular models for studying disease pathogenesis.

To date, iPSCs have been extremely useful for generating key cell types for studying diseases. By comparing specific cell types derived from diseased and healthy iPSCs, various phenotypic changes can be observed, including protein expression, cellular morphology, and function. As iPSCs have been shown to retain the entire genomic and epigenomic background of patients (10), they are purported to replicate the disease processes in humans more closely than other disease models. Furthermore, it is a potential scalable disease modeling solution. Today, a wide variety of protocols varying in terms of cellular origin, reprogramming methods and differentiation methods are available to achieve these models. However, consistency and replicability of results across batches are key to the success of these models.

**FIGURE 1:**
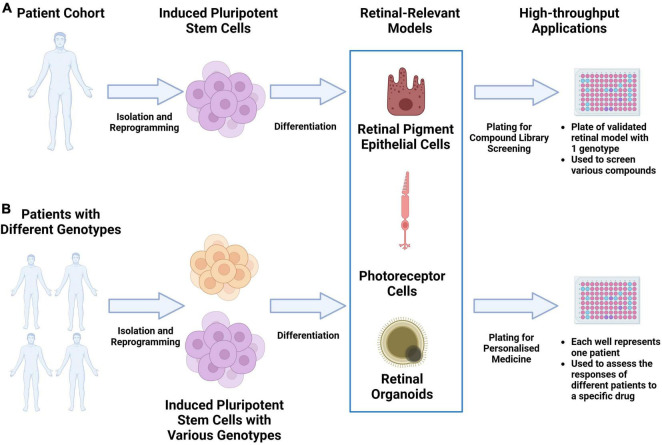
Utilizing iPSC derived retinal models drug discovery and precision medicine workflows. **(A)** Retinal-relevant models can be developed from a specific patient and used for high-throughput screening of various compound libraries. **(B)** Retinal-relevant models can be developed from patients with different genotypes and used to predict the responses of different patients to a specific drug.

As the scalability, consistency and replicability of iPSC-derived disease models continue to be improved, they have the potential to significantly accelerate drug discovery through large-scale drug screening campaigns. A significant cause of clinical-stage drug development failure is the lack of clinical efficacy. This has been attributed partly to the failure of pre-clinical animal models to capture the physiology and pathogenic mechanisms present in human tissue. As described in various reviews, validated iPSC-derived cell types, when structured in a multiarray format, can be used for large compound library screening (11–13). The responses from these iPSC multi-arrays can be used to gauge compound toxicity. If cells derived from multiple iPSCs with different genetic background are used, the responses can be used to identify novel biomarkers of response to facilitate a precision medicine approach. The results from such campaigns can significantly reduce the duration and costs of clinical trials through the appropriate identification of trial candidates. Figure 1 provides an overview of this potential workflow.

This review provides a toolkit for developing iPSC-derived IRD models. Firstly, it will provide an overview of the considerations for developing a robust iPSC-based disease model. Next, a disease-based review of current iPSC-derived IRD models will be done. Finally, technological hurdles and future directions of this field will be described.

## 2 Methodology

To identify the relevant studies involving iPSC-derived retinal cellular models for IRD, a non-systematic literature search was performed in September 2023 in the National Library of Medicine (PubMed) and PubMed Central. The search was based on key words including individual or combination of the following terms: iPSCs, IRDs, RP, LCA, choroideremia, gyrate atrophy, SD, Best’s vitelliform dystrophy. A snowball approach was adopted to identify additional articles that were relevant to the theme. Each article was analyzed based on the inclusion and exclusion criteria to determine their relevance for the narrative review. The inclusion criteria encompassed studies that had mutation data of the patient-derived iPSC models available. Articles that only developed patient-derived iPSCs rather than functionally relevant models were excluded. Publications in non-English language were also excluded.

## 3 Considerations for developing human induced pluripotent stem cell derived disease models

To closely capture the biological features of disease with iPSC technology, considerations such as somatic cell source, reprogramming methods, control strategy, and various cellular differentiation protocols have to be addressed. In this segment, these considerations and the implications on eventual disease models will be discussed.

### 3.1 Somatic cell sources

While the initial iPSC paper published by Yamanaka utilized fibroblasts, today’s iPSC protocols involve adult blood cells, renal tubular epithelial cells, keratinocytes, adipose tissue, and even melanocytes. Each type differs in terms of ease of collection, reprogramming efficiency, and duration. Kim et al. (10) demonstrated that iPSCs obtained through transcription factor-based reprogramming possessed DNA methylation signatures which were characteristic of their somatic origin tissue. These cells also favored their differentiation along lineages associated with the donor cell, which may restrict alternative cell fates (10). Interestingly, these epigenetic marks could also be reset if the cells were differentiated into a type that was different from the somatic cell. iPSC-derived photoreceptor and RPE cell have been successfully generated using dermal fibroblasts, peripheral blood mononuclear cells (PBMCs), renal tubular epithelial cells, and keratinocytes. Further investigations on the effects of epigenetic marks on the fate of these cells should be explored.

#### 3.1.1 Dermal fibroblasts

Dermal fibroblasts are mesenchymal cells located in the dermis of the skin. They produce proteins like laminin and fibronectin to maintain the skin’s extracellular matrix. As the initial iPSC experiments were conducted using fibroblasts, they continue to be a popular choice of somatic cells for the development of protocols. Photoreceptor and RPE cells for disease modeling have been obtained using human fibroblast-derived iPSCs (14). Despite being well-researched, harvesting of this somatic cell source requires a painful procedure of a skin punch biopsy, with risks of bleeding site infections. Hence, other less invasive methods to generate iPSCs have been developed.

#### 3.1.2 Blood cells

Induced pluripotent stem cells can be generated from two main sources of blood cells – adult or umbilical blood. Interestingly, umbilical blood-derived iPSCs have not been widely used for the generation of photoreceptor and RPE cells in disease modeling, probably due to the intricacies of umbilical cord banking, including the need for the banking to be done immediately after birth, good characterization of patient details and follow-up. Hence this review will focus on the use of adult blood instead.

There are two methods of obtaining iPSCs from adult blood. The first method involves administering granulocyte colony stimulating factor to encourage production and mobilization of hematopoietic stem cells from the bone marrow to the peripheral blood. This is followed by apheresis, where the CD34^+^ stem cells can be isolated from the patient’s blood (15). While it has been proven reliable for generation of iPSCs to study cells of the hematopoietic lineage, the apheresis procedure is time-consuming and patients can develop side effects from G-CSF administration, thereby limiting the modality to healthy patients. A less invasive method was subsequently developed which utilized PBMCs, in particular mature T and myeloid cells (16). While less common, iPSCs derived from PBMCs have also been successfully differentiated into RPE cells for interrogation of IRD pathogenesis (17).

#### 3.1.3 Renal tubular epithelial cells (urine)

Induced pluripotent stem cells have also been generated from cells found in patient urine samples (18). These cells are typically exfoliated renal epithelial cells. As compared to the other collection methods, urine is a non-invasive source of iPSCs. Hence, samples are easily obtained including populations such as children and the elderly. Furthermore, the reprogramming efficiency is relatively high, achieving up to 4% when utilizing retroviral delivery of exogenous factors. However, the isolation of these cells from urine has to be done carefully, as the samples can be contaminated by both normal flora and pathogenic bacteria. Normocure (InvivoGen), a broad-spectrum antibiotic, can be administered to such samples without any effect on number of iPSC colonies isolated downstream (19). iPSCs derived from this cell source have also been used for IRD disease modeling through differentiation into organoids (20).

#### 3.1.4 Keratinocytes (hair)

While keratinocytes can be found in the skin, a less invasive way of obtaining these cells is through the harvesting of hair follicles. Hair follicle stem cells, lying in the bulge of the outer root sheath, eventually differentiates into keratinocytes. Due to the importance of this anatomical structure, a key contributor to success of iPSC generation from keratinocytes is the hair follicle harvesting process. The root of the hair requires the outer root sheath attached for further culturing and isolation. Nevertheless, the non-invasiveness of sample collection makes this an attractive source for iPSC generation. As compared to fibroblasts, keratinocytes can be reprogrammed faster and have greater reprogramming efficiency. Three-dimensional retinal organoids have been obtained from keratinocyte-derived iPSCs (21).

### 3.2 Reprogramming methods

Another important factor of the disease modeling workflow is the reprogramming method of source cells. Reprogramming involves the insertion of the four transcription factors, Oct 3/4, Sox2, c-Myc, and Klf4 into the cell. The large variety of reprogramming methods can be broadly divided into integrating and non-integrating methods. These methods have been highlighted and discussed in great detail in several review papers (22–24). The methods can be broadly categorized into integrating and non-integrating methods. Integrating methods utilize viral vectors that integrate into the host’s genome, such as lentiviruses and retroviruses (25–27). These methods typically have high reprogramming efficiency but suffer from the risks of insertional mutagenesis which can affect the validity of disease phenotype being modeled. In non-integrative methods, the inserted material that encodes for the transcription factors reside in the cytosol instead. These methods include utilizing the Sendai RNA virus, transfection of episomal plasmids, delivery of mRNA, and synthetic reprogramming proteins (28–31). In general, non-integrating methods are preferred over integrating methods due to lower rates of unwanted genetic aberrations which can threaten the biological validity of the disease model downstream (32). Nevertheless, the integrating methods are more reliable and efficient for reprogramming. iPSC models for studying IRDs have utilized both types of methods.

### 3.3 Quality control of iPSCs

To standardize the quality of iPSCs produced, the stem cell community encourages banking of iPSCs into large-scale banks. As part of the process, quality control data is required. These banks include the European Bank for Induced Pluripotent Stem Cells (EBiSC) and Human Pluripotent Stem Cell Registry (hPSCReg). While the quality control data requirement may vary slightly, the uniting rationale of banking is to (1) ensure iPSCs generated are from the identified patient, (2) validate pluripotency potential, and (3) identify any factors that may impact the pluripotency potential of generated cells (33).

To validate the identity of iPSCs, next generation sequencing (NGS) methods can be adopted. While sequencing can provide high resolution data, the workflow can be extremely time consuming. A more feasible method is using short tandem repeats (STR) analysis. This involves looking at STR fragments with highly variable lengths and it is based on the principle that unique molecular fingerprints of alleles at different genomic loci are generated. In disease modeling, NGS methods may be worth the additional resources spent as it can also validate the presence of the mutation of interest.

Pluripotency can also be determined in a variety of ways. However, demonstrating the presence of human pluripotency markers (OCT4, NANOG, SSEA-4, and TRA1-60) and the teratoma formation assay are considered the best evidence of pluripotency. The teratoma assay is based on the ability of iPSCs to form tumors derived from all three of the germ layers in immunodeficient mice. However, performing the assay can be time-consuming and technically challenging. Furthermore, there is ongoing discourse regarding the ethical implications of this assay (34).

A wide variety of factors have been associated with abnormal growth and pluripotency potential of iPSCs. These include infections, chromosomal abnormalities, culture conditions and unintended effects of reprogramming methods. In terms of infection, the presence of HIV, HIV2, Hep B, Hep C, and mycoplasma have to be tested. Karyotyping has to be conducted to ensure no chromosomal abnormalities have risen from the induction process. For the culture conditions, the use of surface coating, feeder cells, passage methods, specific media, and rock inhibitor will also have to be declared. Finally, the presence of residual reprogramming materials such as Sendai virus and plasmids will have to be evaluated through polymerase chain reaction as part of the quality control process.

### 3.4 Control strategy

The control strategy for iPSC-derived disease models is important to assure reproducible readouts across repeated experiments. Initial disease modeling studies utilized unaffected cells from family members or even stem-cell banked lines as controls. However, with transcriptional analyses performed at large stem cell repositories, it was noted that genetic differences between individuals were more likely to contribute to phenotypic variances between control and patient-derived lines as opposed to genetic differences between clones from the same patient (35). Today, the use of isogenic controls, cell lines with identical genetic background is preferred to improve the reproducibility of findings.

Isogenic lines can either be created through introduction of specific disease-causing mutations into normal iPSCs or through the correction of patient-derived iPSCs (36), without the introduction of any genetic change. Genomic editing technologies such as the clustered regularly interspaced short palindromic repeats (CRISPR)/Cas9 system can be utilized for this purpose. Choosing between insertion or removal of the mutation will require careful consideration of the mutation. For a highly penetrant mutation, insertion of the mutation into a well-studied control line may be sufficient. However, for an identified variant which has also been found in patients without the disease, but at a lower frequency, significant contribution from the cellular genetic background may be required in order to develop disease. For such instances, removal of the mutation from a patient line and observing for a wild-type phenotype may be more suitable (37).

Another important source of variation is the sex of the iPSC line. It has been shown in early passage that the amount of X-chromosome reactivation may vary significantly. Furthermore, X-chromosome inactivation has also been observed in later passages (38). As a result, when utilizing female iPSC lines, the X-chromosome inactivation status should be reported.

### 3.5 2D and 3D differentiation protocols for disease modeling

One of the earliest protocols for generation of retinal cells from iPSC lines emerged during the late 2000s. The protocol leveraged on the understanding of retinogenesis and earlier work done on embryonic stem cell (ESC) lines (39–42). Utilizing Wnt and Nodal antagonists, human iPSCs in a suspension culture were successfully induced into a heterogenous culture with cells expressing cellular markers of retinal progenitor and RPE cells. These signaling pathways have previously been studied as significant regulators of retinal development (43). When these heterogenous cultures were later treated with retinoic acid and taurine, cellular markers representative of photoreceptors (recoverin and rhodopsin) were also observed (44). Since then, various differentiation protocols have been built upon each other to improve yield and maturity of photoreceptors of RPE for transplantation and disease modeling purposes. These protocols have been thoroughly covered in published reviews (45, 46).

Subsequently, the first protocol for generation of 3D retinal organoids emerged in 2011. Meyer et al. (47) demonstrated that iPSCs could be differentiated into a 3D structure that was similar to the optic vesicle. These vesicle-like structures had physiologically responsive retinal cell types including photoreceptors and RPE cells. From a functional perspective, the photoreceptor-like cells in these vesicular structures demonstrated electrophysiological features of cells undergoing phototransduction (47). These findings led to the development of the 3D retinal organoid field and multiple protocols from independent laboratories have emerged since then. These protocols can be broadly divided into two approaches. The first approach employs a 3D suspension culture throughout, and involves re-aggregating a single cell suspension in ultra-low attachment 96-well plates to form uniform spherical floating embryoid bodies (EBs). Neural induction is then stimulated with Matrigel, which contains basal lamina components, or BMP4. Upon addition of reagents such as N2 and retinoic acid, retinal neuroepithelium will appear at the outer edges of the organoid. As differentiation progresses, a laminar structure may appear (48, 49). In contrast, the second approach combines 2D and 3D techniques (50–53). Ebs of varying sizes are formed from detached human PSC colonies, and neural induction occurs in suspension culture. However, Ebs are then plated into adherent 2D cultures. Following the formation of retinal neuroepithelium, optic vesicles are then excised and isolated to reform 3D organoids. The latter approach has typically achieved better organization and maturation to late-stage organoids with laminated retinal morphology.

Each approach has its own utility. While 2D cultures of photoreceptors or RPE cells do not capture the architecture of the retina, such cultures may be more cost-effective and easier to functionalize into high-throughput assays for drug screening campaigns. However, homogeneity of these cultures is key in drug screening campaigns to ensure the biological response toward the drugs tested is from the cell type of interest. Meanwhile, 3D organoid models, with their superior anatomical and physiological similarities with retinal tissue, allow deep interrogation of retinal biology. For instance, the study of cellular functions which are influenced by the presence of other cell types or even spatial biology aspects of retina-relevant cells. However, 3D organoid models take a long time to develop and also suffer from significant batch variability.

Regardless of the approach chosen, when designing iPSC-derived disease models for phenotypic screens, it may be prudent to adhere to the “phenotypic screening rule of 3” as proposed by Vincent et al. (54). In general, the iPSC-derived disease models should demonstrate (1) high disease relevance with use of appropriate native cell type; (2) a disease relevant stimulus for phenotype production; and (3) an assay readout which correlates with the clinical endpoint of the disease (54). Hence, it may be beneficial to consider the relevant clinical endpoints prior to choosing between a 2D or 3D approach.

## 4 Human induced pluripotent stem cell derived inherited retinal disease models

Ideally, a convincing iPSC-derived IRD model should fulfill the following criteria: (i) include lines derived from multiple patients sharing the same mutation, (ii) compare disease patient-derived cells to a gene-edited version or wild-type line, as well as (iii) an additional age- and sex-matched or sibling control, and, finally, (iv) be able to demonstrate functional rescue of the mutated gene. In practice, it is not always feasible to attain this level of control in every model, and a study should not be disregarded if it does not meet all of these criteria. In the following segments, the various available *in vitro* disease models of RP, LCA, choroideremia, gyrate atrophy, SD, and Best’s vitelliform dystrophy will be covered.

### 4.1 Retinitis pigmentosa

Retinitis pigmentosa is the most common form of inherited retinal dystrophy, with a prevalence of approximately 1:4,000 (55). It begins with night blindness and loss of peripheral visual field, and can progress to legal blindness. It is characterized by high genetic heterogeneity, with over 130 genes reported to cause either isolated (approximately 90 genes) or syndromic forms (approximately 40 genes). The majority of RP genes follow an autosomal recessive inheritance, followed by autosomal dominant and X-linked patterns. While bony-spicule hyperpigmentation of the peripheral retina is a common finding in the disease, the cellular and molecular mechanisms underlying this phenotype remain unclear.

Induced pluripotent stem cell disease modeling was first used to identify novel RP mutations. Tucker et al. (56) validated a pathogenic Alu insertion into male germ cell-associated kinase (MAK) in 2D patient-derived retinal progenitor cells. The insertion led to cells having a MAK transcript that was lacking in exon 9 and 12 in comparison with the control.

With the refinement of iPSC differentiation protocols into 2D or 3D retinal cultures, early studies utilizing patient-derived iPSCs to model RP sought to demonstrate degenerative features suggestive of a pathological phenotype, such as photoreceptor cell death or oxidative stress. The Takahashi group was the first to successfully derive patient-derived cell lines associated with four known causative mutations in axonemal microtubule associated protein (RP1), Pim-1 kinase associated protein (RP9), peripherin 2 (PRPH2), and RHO (57), and subsequently showed that 2D rod photoreceptors harboring a RHO mutation underwent degeneration *in vitro* and expressed markers of oxidation and endoplasmic reticulum stress (57, 58). Other groups have similarly focused on demonstrating cellular stress caused by pathogenic mutations (21, 59–61). Additionally, the use of reagents that could modify ER stress-related pathways were also shown to reduce apoptosis in 2D rod photoreceptors harboring mutant rhodopsin (59).

However, the generation of mature photoreceptors from 2D protocols remained a challenge, and efforts in the following years were directed toward the efficient differentiation of hiPSCs in multilayer 3D retinal organoids, with great success. Subsequent studies were therefore able to additionally demonstrate cell-specific changes in gene expression and morphology. For example, Guo et al. (62) derived an iPSC line from the urine cells of an RP patient with a novel pathogenic USH2A mutation, and generated 3D retinal organoids including RPE. These showed early developmental abnormalities, including reduced laminin expression during retinal organogenesis, abnormal retinal neuroepithelium differentiation and polarization, as well abnormal RPE morphology and increased apoptosis (62).

Increasingly sophisticated 3D differentiation protocols have allowed a closer recapitulation of neural retina development and maintenance, which in turn has facilitated interrogation of the pathogenic mechanisms underlying various RP mutations. For example, in X-linked RP (XLRP), mutations in the Retinitis Pigmentosa GTPase Regulator (RPGR) gene account for 70%–90% of XLRP and 10%–15% of all RP (63). Megaw et al. (64) showed that *RPGR*-mutant, iPSC-derived 3D photoreceptors displayed abnormally increased actin polymerization and defective rhodopsin trafficking within photoreceptor connecting cilium, which was due to defective activation of the actin-severing protein gelsolin by RPGR. Expression of activated gelsolin was shown to rescue the ciliogenic defects observed in RPGR-depleted RPE cells (64). Further studies by the Jin group, *RPGR*-mutant, iPSC-derived 3D retinal organoids showed significant defects in photoreceptor morphology, localization, transcriptional profiling, and electrophysiological activity, as well as RPE and photoreceptor ciliopathy. CRISPR/Cas9-mediated correction was able to rescue photoreceptor structure and electrophysiology, reverse the observed ciliopathy, and restore gene expression comparable to healthy controls using transcriptome-based analysis (20). Most impressively, the Jin group recently established the first late-onset RP model using retinal organoids generated from late-onset RP patient-derived iPSCs harboring a *PDE6B* mutation, which demonstrated markedly changed gene expression profile compared to wild-type by transcriptomic analysis. Of note, changes in the expression of genes regulating cGMP hydrolysis led to high levels of cGMP, which led to impaired formation of synaptic connections and the connecting cilium in photoreceptor cells (65). In a more recent study, Chahine Karam et al. (66) probed the pathogenicity of a novel intronic RPGR variant with retinal organoids. Mutant organoids demonstrated abnormal RNA splicing, reduced RPGR expression, loss of localization of RPGR in the photoreceptor cilium, mislocalization of rhodopsin and increase photoreceptor apoptosis (66).

Other studies have also examined pathological RP mutations affecting RPE instead of photoreceptors only. Li et al. (67) demonstrated actin disorganization in iPSC-derived RPE harboring membrane frizzled-related protein (MFRP) mutations. Gene therapy with AAV8-MFRP then rescued the actin disorganization phenotype, restored apical microvilli, and recovered pigmentation and transepithelial resistance (TER) (67). Other groups looked at causative mutations in MER receptor tyrosine kinase (MERTK), which results in phagocytic defects in patient-derived RPE (68, 69). Translational read through inducing drugs (TRIDs) then restored MERTK and reinstated phagocytosis function in RPE with mutant *MERTK* (69). Other groups have similarly demonstrated the effectiveness of TRIDs in rescuing other nonsense mutations that lead to premature termination codons (70, 71).

More recently, the Lako group generated transcriptomic profiles from pre-mRNA processing factor 31 (PRPF31)-mutated patient-derived retinal organoids and RPE, which revealed that mis-splicing of genes implicated in ciliogenesis and cellular adhesion occurred only in patient-specific retinal cells. This was associated with severe RPE defects, including disrupted polarity, reduced TER, impaired phagocytosis, and decreased cilia length and counts. Disrupted cilia morphology was also seen in patient-derived photoreceptors, and was associated with progressive degeneration and cellular stress. Encouragingly, CRISPR/Cas9-mediated correction was able to rescue both molecular and cellular phenotypes in RPE and photoreceptors (72). Future iPSC-derived models of IRDs will likely be expected to similarly demonstrate molecular and cellular phenotypes, as well as functional rescue of the mutated gene with corresponding phenotypic rescue.

Given that RP is the most common IRD, it is also the most widely studied in terms of pathophysiology. Despite this, developing a robust disease model that can capture the genotype-phenotype correlation for RP has still been challenging. This is even with utilizing iPSC-derived cellular models, where the genetic predisposition to disease is assumed to be captured. Firstly, the development of RP is increasingly recognized to be significantly influenced by epigenetic changes (73, 74). Epigenetic changes can arise due to environmental factors such as changing oxygen and glucose levels. As cell culture conditions may differ significantly compared to the human retina, the impact of such conditions on iPSC-derived cellular models of RP should be studied. Secondly, genetic heterogeneity is a significant confounder of establishing genotype-phenotype correlation (75). This can arise as genetic, allelic, phenotypic or clinical heterogeneity. In particular, for iPSC-derived disease models, developing a robust control strategy can be challenging when various genes provoke the same disease phenotype. Finally, it has been suggested that current 3D retinal organoid differentiation protocols are limited in terms of development up to 38 weeks (76). These models may not be mature enough to fully demonstrate the changes associated with the disease. This is especially so for patients with late-onset RP, which can arise in the late 30s. Further development of these protocols to produce more matured cells may potentially unveil novel mechanisms underpinning this form of RP.

### 4.2 Leber congenital amaurosis

Leber congenital amaurosis is a rare retinal dystrophy affecting approximately 3 in 100,000, and can be inherited in autosomal recessive and dominant patterns. It is the most severe retinal dystrophy, causing blindness by the age of 1 year. Patients usually present with nystagmus, sluggish pupillary responses, severely decreased visual acuity, and photophobia.

One of the earliest models for LCA used fibroblasts derived from two patients with unidentified mutations to generate 2D neurons and RPE, and identified differentially expressed genes (77). Subsequent work focused on two genes, which are the most common cause of LCA: centrosomal protein 290 kDa (CEP290), and RPE-specific 65 kDa (RPE65). Early work in fibroblasts from four patients with CEP290 mutations showed defects in ciliogenesis, which was rescued by lentiviral-mediated transduction of CEP290 (78). These findings were reproduced in 3D retinal organoids by two other groups (79, 80), one of which demonstrated functional rescue using antisense oligonucleotides to CEP290 (80). Another group looking at CEP290 mutations in patients with Joubert syndrome found that CEP290 mutations cause RPE maturation defects as well, which precede photoreceptor degeneration (81).

Like with RP, iPSC disease modeling has also been used to identify novel pathogenic mutations in LCA, particularly in RPE65 (82, 83). This is increasingly important also for assessing patient eligibility for participation in gene augmentation trials, which are currently ongoing for RPE65. Aryl hydrocarbon receptor-interacting protein-like 1 (AIPL1) and IQ calmodulin-binding motif containing B1 (IQCB1) are some of the recent additions to the iPSC-derived organoid models for LCA (68, 84). In the latter study, photoreceptor ciliopathy defects were rescued by AAV2-mediated NPHP5 gene replacement (84).

A classical finding of most LCA cases is the greatly reduced or absent full-field electroretinogram (ERG) waveforms. Many of the genes associated with LCA are involved in the phototransduction cascade (85). The loss of these protein functions results in the failure of the visual cycle and eventual loss of the ERG waveforms. As majority of 3D retinal organoid protocols produce photoreceptors with attenuated outer segments, they are unable to fully replicate the phototransduction process. In the comparisons of patch clamp results of retinal organoid and non-human primate photoreceptors, it was noted that organoid light responses took a longer time to peak and typically had smaller amplitudes. Attenuated light responses may not be able to capture the pathologic effects of LCA-associated mutations. As organoid differentiation protocols continue to improve, future disease modeling efforts of LCA can attempt to utilize this method to assess the electrophysiological implications of the LCA mutations (86).

### 4.3 Choroideremia

Choroideremia is a rare X-linked inherited chorioretinal dystrophy affecting 1 in 50,000–100,000 individuals. Choroideremia tends to become symptomatic in the first decade of life, starting with nyctalopia then progressing to peripheral vision loss; central vision tends to be spared till the fifth decade of life onward, when rapid deterioration is common. In contrast to the bony-spicule pigment clumping seen in RP, choroideremia patients tend to manifest first with diffuse pigment clumping, followed by retinal atrophy with baring of the underlying sclera and choroidal vessels.

The disease is caused by mutations in the CHM gene that encodes Rab escort protein 1 (REP1), which is essential for intracellular vesicular trafficking in photoreceptors and RPE. There are over 110 known mutations in CHM, all of which result in loss-of-function of REP1. REP1 causes efficient prenylation of Rab proteins to prevent vesicular trafficking defects. This protects the cell against oxidative stress and aids in nutrition and waste management of both photoreceptor and RPE cells.

To date, only RPE-based models are available. These were inspired by the biochemical functions of REP1. Cereso et al. (87) demonstrated that iPSC-derived RPE cells with CHM mutations had a higher proportion of unprenylated Rab proteins and, in particular, a larger proportion of Rab27a were located in the cytosol instead of the cellular membrane (87). This phenotype was rescued when a AAV2/5-mediated delivery of the CHM gene was administered. More recently, Duong et al. (17) also demonstrated a reduction of phagolysosomal activity across four different mutant iPSC-derived RPE cell lines when they were exposed to pHrodo *Escherichia coli* BioParticles. Similarly, these phenotypes were rescued when treated with recombinant AAV7m8.hCHM (17).

To date, it still remains unclear if photoreceptors degenerate autonomously in choroideremia pathogenesis or as a result of abnormal RPE function. Histopathological studies have been conflicting, with some highlighting the RPE (88) and others highlighting photoreceptors as the origin of pathology (89). An *in vitro* model that is able to capture the interactions between photoreceptors, RPE and choroid will likely shed more insight into the pathogenesis mechanisms. However, in current organoid differentiation protocols, these three components are not spatially associated with each other. Future protocols that are able to generate such organoids will be able to demonstrate the interplay of cells during the degenerative process and potentially highlight a cellular population that should be targeted for therapeutic effect.

### 4.4 Gyrate atrophy

Gyrate atrophy is another rare autosomal recessive inherited chorioretinal dystrophy, and is due to mutations in the ornithine aminotransferase (OAT) gene. More than 50 variants have been identified, with missense mutations occurring most frequently, all of which result in truncation of the enzyme OAT.

This causes protein degradation and a consequent increase in plasma ornithine concentrations, leading to chorioretinal degeneration. It has been suggested that the RPE is the first site of insult due to its reliance on OAT activity for metabolic functions.

An early study using an iPSC disease model derived from the fibroblasts of a patient with gyrate atrophy revealed that although diseased iPSCs carry a significant mutational load at initial isolation, the clonal events and prolonged culture required for correction of the pathogenic mutation via homologous recombination could be accomplished without a significant increase in mutational burden (90). This was indeed a significant first step in the direction of using gene-corrected iPSCs in transplantation medicine for gyrate atrophy. Further work by the same group successfully demonstrated functional rescue using a simple colorimetric assay for measuring OAT activity (50, 91). Patient-derived iPSC cells harboring an A226V OAT mutation were differentiated into 3D optic vesicle-like structures and RPE, which exhibited a functional defect that could be corrected both by pharmacological means (vitamin B6) or targeted gene repair via homologous recombination (47).

While ornithine has been identified to be the etiological agent of gyrate atrophy, the pathophysiologic mechanism contributing to progressive retinal degeneration is not well understood. This is partly due to the rarity of the disease and lack of human retinal samples for study. To date, there has only been 1 post-mortem, histopathology study of a patient with gyrate atrophy. As the patient was 92 years of age, the histopathology changes on the retina such as photoreceptor atrophy and hyperplastic adjacent RPE were reflective of late-stage gyrate atrophy. However, the study was unable to provide any insights regard the chronology of changes associated with the retina during OAT deficiency (92). There have been attempts to replicate gyrate atrophy in mice models. While these models respond significantly to arginine-restricted diets that control ornithine levels, such diets do not always slow the progression of retinal degeneration in patients (93). This suggests the need for further investigation into the mechanisms of ornithine toxicity. Future studies should build on these findings to better understand the origin of ornithine and its toxicity mechanisms.

### 4.5 Stargardt disease

Stargardt disease is an autosomal recessive inherited macular dystrophy affecting 1 in 8,000–10,000 (2). Majority of affected individuals have a mutation in the photoreceptor-specific ATP binding cassette subfamily A member 4 (*ABCA4*) gene, which encodes for a protein responsible for retinoid metabolism in the retina. ABCA4 dysfunction causes an accumulation of all-trans and 11-cis retinoids in photoreceptors, eventually forming A2E, which is toxic to both photoreceptor and RPE cells. Between 7% and 30% of STGD1 patients carry only a single ABCA4 mutation (94–96). SD is characterized clinically by three diagnostic clinical findings: (1) macular affection – whereby visual dysfunction and retinal architecture degeneration typically originates in the central macula; (2) fundus flecks – which are lipofuscin aggregates, a lipid byproduct of a dysfunctional visual cycle; and (3) peripapillary sparing of the retina of the pathognomonic features especially in the early stages of the disease.

To date, multiple groups have achieved well-characterized iPSCs from patients with STGD1 (97, 98). Recent work by Sangermano et al. (99) examined 17 patients sharing a common variant in ABCA4 (c.5461-10T→C), and derived three iPSC lines and one control line. mRNA analysis of the iPSC-derived 2D photoreceptor progenitors revealed mRNA exon skipping (99). This finding was reproduced in fibroblasts obtained from patients harboring the same variant (100).

While SD is traditionally believed to affect the photoreceptors first before the toxic byproducts affect RPE cells through outer segment phagocytosis, recent observations have suggested the possibility of lipid-handling defects contributing to the pathogenesis of the disease. This is independent of the disease process in photoreceptor cells. The work by Farnoodian et al. (101) examined the impact of the ABCA4 (c.6088C>T) mutation on lipid handling in iPSC-derived RPE cells. When fed with photoreceptor outer segments, mutant RPE cells demonstrated an increase in ceramide (lipid metabolite) accumulation at the apical membrane of RPE cells. Furthermore, mutant RPE cells also had a 50%–70% reduction in POS digestion rate compared with control cells. These results suggest that loss of function of ABCA4 can disrupt lipid homeostasis in RPE cells, resulting in toxic byproducts and eventual cell death that is independent of photoreceptor death (101).

While considerable progress has been made on the molecular characterization of ABCA4 and its role in the visual cycle, significant questions remain. From a biochemical perspective, while the substrate binding domains have been fully elucidated, the functions of the regulatory domains of the ABCA4 proteins are still not fully understood (102). While iPSC-derived models can be used as a source of mutant proteins, the downstream methods for protein function evaluation are complex. A key assay which helped elucidate the function of the substrate-binding domain is the N-retinylidene-phosphatidylethanolamine (N-ret-PE) assay. Unfortunately, this requires high-performance liquid chromatography and mass spectrometry, which can be challenging to be converted to high-throughput for drug screening purposes (103). From a cellular perspective, the mechanisms behind photoreceptor and cellular death still needs to be further defined. It has been suggested in age-related macular degeneration studies that bis-retinoid constituents can trigger complement systems, resulting in RPE atrophy (104). However, this has yet to be studied in the context of ABCA4 mutations. As organoid differentiation protocols become more complex, the interplay between photoreceptors, RPE and immune cells can be further studied to fully uncover the disease mechanisms of SD.

### 4.6 Best’s vitelliform dystrophy

Best’s vitelliform dystrophy, or Best disease, is an autosomal dominant disease affecting as many as 1 in 18,000, and is caused by mutations in the RPE-specific calcium-activated chloride channel Bestrophin 1 (BEST1). Mutations in the *BEST1* gene causes five clinically recognized retinal dystrophies, collectively referred to as bestrophinopathies, of which Best’s vitelliform dystrophy is the most common. The first Best disease iPSC model examined point mutations (A146K and N296H) in two patients, and showed that patient-derived RPE displayed disrupted fluid flux and photoreceptor outer segment handling compared to sibling controls (105). Subsequent work by the same group showed that these two mutations also caused delayed photoreceptor outer segment degradation by RPE, which could be rescued by valproic acid and rapamycin (106). Other independent groups examined various other point mutations, and similarly showed a reduction in anion-channel mediated currents (107–109). One such study looking at the point mutations P274R and I201T was able to demonstrate rescue of Cl− current cursing baculoviral vectors carrying wild-type BEST1 (109). Another more recent study looking at two separate point mutations R141H and I366fsX18 once again confirmed delayed photoreceptor outer segment degradation by RPE, and concluded that the phenotypic findings were not wholly due to nonsense-mediated decay of the mutant protein, nor the complete absence of Best1 protein (110).

Induced pluripotent stem cell-derived Best disease models have managed to replicate many of the pathogenic processes found in RPE cells. However, the lack of a macula in these models may still pose a limit to how much these models can help in elucidating the pathogenic mechanisms of Best disease. The classical presentation of Best’s vitelliform dystrophy is the egg-yolk lesion noted at the macula and sparing of the peripheral retina. Given that the RPE cells vary in shape, size and appearance based on the location of the retina, gene expression and cellular function may vary as well (111). If newer differentiation protocols can eventually produce organoids with macula-like structures, these models may help explain these macular signs seen clinically (112).

## 5 Technology hurdles and future directions

Induced pluripotent stem cell-based disease modeling allows the development of human tissue that captures the underlying genetics of disease. It is exceptionally powerful for the study of retinal diseases where human samples are extremely difficult to obtain due to the lack of accessibility and regenerative capabilities. However, there are still various hurdles for the technology to overcome in order to unlock its full potential.

### 5.1 Defining differentiation efficiency and cellular maturity

Induced pluripotent stem cells, given their pluripotent nature, usually give rise to a heterogenous culture when differentiated. The ideal differentiation protocol should have a high efficiency for generating retinal relevant cell types such as photoreceptors, RPE and ganglion cells. This avoids the studying of other cellular populations which are not relevant to the pathophysiology of the disease. The cells should also be mature and resemble their counterparts both structurally and functionally in the human eye.

In majority of studies, cellular identity is usually determined through a combined assessment of morphology, expression of signature proteins and genes associated with the cell type. These evaluation criteria are usually inspired by findings from fetal studies of retinogenesis and histopathology studies of the human eye. For instance, commonly used proteins and genes for photoreceptors include recoverin, RHO, and CNG. Meanwhile, RPE-relevant proteins and genes include ZO-1, RPE65, and CRALBP. A detailed description of these markers can be found in available reviews (113). However, due to the lack of guidelines, majority of characterization attempts to demonstrate a subset of the available cellular markers. The rationale for an incomplete expression of cellular-specific proteins is also not well-studied. Furthermore, the methods for demonstrating expression have varied widely from immunofluorescence staining, Western blotting, flow cytometry, qPCR, or RNA-seq. This lack of consensus has made it challenging to fully evaluate the differentiation efficiency of various protocols, especially if non-quantitative methods have been utilized. Future efforts in the field should drive toward a consensus such that disease modeling efforts can be compare and contrasted easily.

Functional maturity of the developed 2D or 3D culture is essential for disease modeling. However, not all studies have demonstrated such assessments (Tables 1–6). For instance, phototransduction is a key function of the photoreceptor cell. While it is implicated in various IRDs, such as LCA and RP, many studies have not shown electrophysiological characterization results. This could potentially be due to the lack of simple methods for characterization. For instance, patch clamping and multi-electrode arrays which are typically utilized for this are highly specialized techniques that not many cellular facilities have access to. In recent times, optogenetic-based technologies that enable optical methods of assessing electrophysiology have emerged (114). While these studies have been predominantly in neurons, harnessing such technologies to study the retina may pave the way forward to an easier and more standardized functional assessment of photoreceptors.

**TABLE 1:** iPSC-derived models of retinitis pigmentosa.

Publication	Gene; mutation	Number of patients	iPSC source	Re-programming method	Culture type	Control iPSC	Rescue	Relevant readouts
Modeling retinal degeneration using patient-specific induced pluripotent stem cells (57)	1. Axonemal microtubule associated (RP1); 721Lfs722X 2. Pim-1 kinase associated protein (RP9); H137L 3. Peripherin 2 (PRPH2); W316G 4. Rhodopsin (RHO); G188R	5	Fibroblast (skin biopsy)	Retroviral transduction	2D, photoreceptors (rods)	Wild-type	No	Mutant rod cells: 1. Undergo degeneration *in vitro* 2. Express markers of oxidation and endoplasmic reticulum (ER) stress 3. Exhibit differences in response to antioxidants depending on their RP genetic subtype
Integration-free induced pluripotent stem cells derived from retinitis pigmentosa patient for disease modeling (58)	RHO; G188R (exon 3 c.562G>A)	1	Fibroblast (skin biopsy)	Non-integrating Sendai virus	2D, photoreceptor (rods), RPE	Wild-type	No	Mutant rod cells: 1. Undergo degeneration *in vitro* 2. Express markers of oxidation and endoplasmic reticulum (ER) stress
Exome sequencing and analysis of induced pluripotent stem cells identify the cilia-related gene male germ cell-associated kinase (MAK) as a cause of retinitis pigmentosa (56)	Male germ cell-associated kinase (MAK); homozygous Alu insert in exon 9	2	Fibroblast (skin biopsy)	Lentiviral transduction	2D, retinal progenitor cells	Wild-type and non-MAK RP iPSC control	No	Alu insertion in exon 9 of MAK prevents normal switch to retina-specific isoform of MAK which contains exon 9 and 12
Patient-specific iPSC-derived photoreceptor precursor cells: a means to investigate retinitis pigmentosa (21)	Usherin (USH2A); IVS40 and Arg4192His	2	Keratinocytes	Non-integrating Sendai virus	3D, retinal organoids	Wild-type and MAK-associated RP iPSC control	No	Mutant rod cells: 1. Undergo degeneration *in vitro* 2. Integrate and differentiate into morphologically and immunohistochemically recognizable photoreceptors post-transplantation into *Crb1* mutant mice
The use of induced pluripotent stem cells to reveal pathogenic gene mutations and explore treatments for retinitis pigmentosa (59)	RHO; E181K	1	Fibroblast (skin biopsy)	Retroviral transduction	2D, photoreceptor (rods)	Wild-type	Yes, helper-dependent adenoviral vector (HDAdV) gene transfer	Mutant rod cells display reduced survival, elevated ER-stress and apoptosis markers
Gene therapy in patient-specific stem cell lines and a preclinical model of retinitis pigmentosa with membrane frizzled-related protein defects (67)	Membrane frizzle-related protein (MFRP); IVS10+5G>A, 492C (1-bp del)	2	Fibroblast (skin biopsy)	Lentiviral	2D, RPE	Wild-type	Yes, AAV8 (Y733F) gene transfer	Mutant RPE display actin disorganization phenotype
Translational read-through of the RP2 Arg120stop mutation in patient iPSC-derived retinal pigment epithelium cells (70)	ARL3 GTPase activating protein (RP2); c.519C>T (p.R120X)	1	Fibroblast (skin biopsy)	Non-integrating episomal vectors	2D, RPE	Healthy, sex-matched control	Yes, translational read through inducing drugs (TRID) G418 and PTC124	Mutant RPE display phenotypic defects in IFT20 localization, Golgi cohesion and Gb1 trafficking
Arl3 and RP2 regulate the trafficking of ciliary tip kinesins (71)	ARL3 GTPase activating protein (RP2); c.519C>T (p.R120X)	1	Fibroblast (skin biopsy)	Non-integrating episomal vectors	3D, retinal organoids	Healthy, sex-matched control	Yes, TRID	Mutant photoreceptor cells display reduced Kif7 staining at cilia tips compared to control
Human iPSC derived disease model of MERTK-associated retinitis pigmentosa (130)	MER receptor tyrosine kinase (MERTK); Ser331 Cysfs*5	1	Fibroblast (skin biopsy)	Sendai virus	2D, RPE	Healthy control	No	Mutant RPE display defective phagocytosis
Rescue of the MERTK phagocytic defect in a human iPSC disease model using translational read-through inducing drugs (69)	MERTK; 61+1G>A, 1951C>T; biallelic	1	Fibroblast (skin biopsy)	Episomal plasmid vectors	2D, RPE	Wild-type	Yes, TRID (G418 and PTC124)	Mutant RPE: 1. Express low levels of *MERTK* mRNA and undetectable *MERTK* protein 2. Display defective phagocytosis
Patient-specific induced pluripotent stem cells to evaluate the pathophysiology of TRNT1-associated retinitis pigmentosa (60)	tRNA nucleotidyl transferase, CCA-adding 1 (TRNT1), various indels	3	Fibroblast (skin biopsy)	Non-integrating Sendai virus	3D, retinal organoids	Healthy, age-matched control	No	Mutant retinal organoids: 1. Express reduced levels of full-length TRNT1 protein and a truncated smaller protein 2. Exhibit a deficit in autophagy, as evidenced by aberrant accumulation of LC3-II and elevated levels of oxidative stress
Gelsolin dysfunction causes photoreceptor loss in induced pluripotent cell and animal retinitis pigmentosa models (64)	RPGR deletion; g.ORF15+689-692del4	2	Fibroblast (skin biopsy)	Lentiviral transduction	3D, retinal organoids	Healthy, sex-matched control	Yes, overexpression of activated gelsolin	Mutant retinal organoids: 1. Display abnormal actin polymerization 2. Display Rhodopsin mislocalization
Gene correction reverses ciliopathy and photoreceptor loss in iPSC-derived retinal organoids from retinitis pigmentosa patients (20)	RPGR; frameshift mutations c.1685_1686delAT in exon 14, c.2234_2235delGA, and c.2403_2404delA in ORF15	3	Urinary cells, fibroblasts	Lentiviral transduction; plasmid vectors	3D, retinal organoids and RPE	Healthy wild-type control	Yes, CRISPR-Cas9	Mutant retinal organoids: 1. Display decreased photoreceptor cell numbers 2. Display significantly abnormal rod and cone photoreceptor morphology, with dislocation of opsins 3. Express lower levels of genes regulating photoreceptor maturation 4. Express higher levels of necrosis and inflammation receptors 5. Display GFAP labeling of Muller cells throughout entire retina thickness 6. Display decreased expression of hyperpolarization-activated cyclic nucleotide-gated (HCN-1) channels and impaired hyperpolarization-activated potassium current 7. Display shortened photoreceptor cilia Mutant RPE display shortened RPE cilia
Disrupted alternative splicing for genes implicated in splicing and ciliogenesis causes PRPF31 retinitis pigmentosa (72)	Pre-mRNA processing factor 31 (PRPF31); c.1115_1125del11, c.522_527+10del	4	Fibroblast (skin biopsy)	Non-integrating Sendai virus	3D, retinal organoids and RPE	Healthy wild-type controls	Yes, CRISPR-Cas9	Mutant RPE: 1. Display disrupted apical–basal RPE polarity 2. Reduced trans-epithelial resistance and phagocytic capacity 3. Decreased cilia length and incidence Mutant photoreceptors display disrupted cilia morphology in photoreceptors, associated with progressive degeneration and cellular stress
Modeling retinitis pigmentosa : retinal organoids generated from the iPSC of a patient with the USH2A mutation show early developmental abnormalities (62)	USH2A; c.8559-2A>G/c.9127_9129delTCC	1	Urine cells	Non-integrating Sendai virus	3D, retinal organoids and RPE	Healthy age- and sex-matched controls	No	Mutant retinal organoids: 1. Display lower laminin expression 2. Display abnormal retinal neuroepithelium differentiation and polarization 3. Display abnormal RPE morphology and increased apoptosis 4. Express lower levels of cilium-associated and dopaminergic synapse-related genes
Patient-specific retinal organoids recapitulate disease features of late-onset retinitis pigmentosa (65)	Rod cGMP-phosphodiesterase type 6 (PDE6B); c.694G>A	1	Peripheral blood mononuclear cells	Episomal plasmid vectors	3D, retinal organoids	Healthy control	No	Mutant retinal organoids express significantly higher cGMP levels that lead to impaired formation of synaptic connections and connecting cilium in photoreceptors
Molecular pathology of Usher 1B patient-derived retinal organoids at single cell resolution (61)	Class 7 myosin (MYO7A); c.6070C>T and c.223G>C; c.1996C>T and c.133-2A>G	3	Fibroblast (skin biopsy)	Non-integrating Sendai virus	3D, retinal organoids	Healthy control	No	Mutant retinal organoids: 1. Do not display degenerative features (normal size, ONL thickness, photoreceptor marker expression) at fetal retina-equivalent stage, consistent with the onset of USH1B in childhood 2. Display increased aberrantly expressed genes over time 3. At 35 weeks, display adaptive responses to stress in rods (but not cones), and enrichment for apoptotic signaling pathways in Muller cells
Human iPSC-derived retinal organoids and retinal pigment epithelium for novel intronic RPGR variant assessment for therapy suitability (66)	RPGR, c.1415-9A>G	1	Fibroblasts (skin biopsy)	Episomal plasmids	3D, retinal organoids and RPE	Healthy control	No	Mutant retinal organoids: 1. Abnormal RNA splicing 2. Reduced RPGR expression 3. Loss of localization of RPGR in photoreceptor cilium 4. Mislocalization of rhodopsin 5. Increased photoreceptor apoptosis

**TABLE 2:** iPSC-derived models of Leber congenital amaurosis.

Publication	Gene; mutation	Number of patients	iPSC source	Re-programming method	Culture type	Control iPSC	Rescue	Relevant readouts
Human induced pluripotent stem cells as a tool to model a form of Leber congenital amaurosis (77)	GUCY2D c.154 G>T	2	Fibroblast (skin biopsy)	Retroviral transduction	2D, RPE	Wild-type	No	Mutant RPE had differentially expressed genes. 1. Upregulation of *NNAT* 2. Downregulation of *GSTT1, TRIM61*, and *ZNF558*
CEP290 gene transfer rescues Leber congenital amaurosis cellular phenotype (78)	Centrosomal protein 290 kDa (CEP290); IVS26 (c.2991 + 1655 A>G), Val247 del1gT, Thr835 del2acAG, 6277delG	4	Fibroblast (skin biopsy)	Lentiviral transduction	2D, fibroblast	JK1 fibroblast-like control	Yes, lentiviral transduction	Mutant fibroblasts had impaired ciliogenesis (number of ciliated cells)
Using patient-specific induced pluripotent stem cells to interrogate the pathogenicity of a novel retinal pigment epithelium-specific 65 kDa cryptic splice site mutation and confirm eligibility for enrollment into a clinical gene augmentation trial (82)	Retinal pigment epithelium-specific 65 kDa, retinoid isomerohydrolase (RPE65); L408P, IVS3-11A>G	1	Fibroblast (skin biopsy)	Non-integrating Sendai virus	2D, RPE	Healthy, sex-matched control	Yes, overexpression of gelsolin	Mutant RPE cells had a splicing abnormality involving exonification of 10 nucleotides of intron 3, a translational frame shift and insertion of premature stop codon 9 amino acids downstream
Identification and correction of mechanisms underlying inherited blindness in human iPSC-derived optic cups (80)	CEP290; IVS26 (c.2991+1665A>G)	1	Fibroblast (skin biopsy)	Episomal plasmid vector, electroporation	3D, retinal organoids	Wild-type	Yes, antisense oligonucleotide	Mutant RPE and organoid photoreceptor cells: 1. Aberrant splicing 2. Impaired ciliogenesis (number of ciliated cells and length of cilia)
*In vitro* modeling using ciliopathy-patient-derived cells reveals distinct cilia dysfunctions caused by CEP290 mutations (79)	CEP290; IVS26+1665A>G, c.2501A>G; p.Gln834Arg (variation in KIF7) and c.2882G>A (p.Arg961His change in NPHP4)	6	Fibroblast (skin biopsy)	Non-integrating Sendai virus	3D, retinal organoids	4 healthy controls	No	Mutant fibroblasts (CEP290-JSRD) had aberrant ciliogenesis (number of ciliated cells and length of cilia) Mutant photoreceptor (LCA and JSRD) cells in organoids had ciliogenesis defects
Primary cilium mediated retinal pigment epithelium maturation is retarded in ciliopathy patient cells (81)	CEP290; c.2495_2512, c.5668 G>T (biallelic)	1	Fibroblast (skin biopsy)	Non-integrating Sendai virus	2D, RPE	Healthy sibling control	No	Mutant RPE demonstrated defective primary cilia structures
Generation and characterization of induced pluripotent stem cells and retinal organoids from a Leber’s congenital amaurosis patient with novel RPE65 mutations (83)	RPE65; c.200T>G (p.L67R) and c.430T>C (p.Y144H)	1	Urine cells	Episomal plasmid vectors	3D, retinal organoids and RPE	Healthy control	No	Mutant RPE had lower expression of RPE65 mRNA and protein transcripts
Retinal organoids derived from hiPSCs of an AIPL1-LCA patient maintain cytoarchitecture despite reduced levels of mutant AIPL1 (68)	Aryl hydrocarbon receptor-interacting protein-like 1 (AIPL1); c.265T>C (p.Cys89Arg)	1	Fibroblasts (skin biopsy)	Non-integrating Sendai virus	3D, retinal organoids	Healthy control	No	Mutant organoids had reduced expression of mutant AIPL1 and PDE6 proteins
*In vitro* modeling and rescue of ciliopathy associated with IQCB1/NPHP5 mutations using patient-derived cells (84)	IQ calmodulin-binding motif containing B1 (IQCB1/NPHP5); c.421_422delTT (p.F141fsX6), c.1036G>T (p.E346X), c.1516_1517delCA (p.H506fsX13)	4	Fibroblasts (skin biopsy)	Non-integrating Sendai virus	3D, retinal organoids and RPE	Healthy familial controls	Yes, AAV2	Mutant RPE had abnormal cilia morphology Mutant organoids: 1. Reduced expression of CEP290 protein 2. Rhodopsin mislocalization in photoreceptor cells

**TABLE 3:** iPSC-derived models of choroideremia.

Publication	Gene; mutation	Number of patients	iPSC source	Re-programming method	Culture type	Control iPSC	Rescue	Relevant readouts
Proof of concept for AAV2/5-mediated gene therapy in iPSC-derived retinal pigment epithelium of a choroideremia patient (87)	CHM REP1; 97 bp deletion between duplication of sequence in intron 7	1	Fibroblast (skin biopsy)	Lentiviral transduction	2D, RPE	Healthy controls	Yes, AAV2/5	Mutant RPE: 1. Higher proportion of unprenylated Rab proteins 2. Higher proportion of cytosolic vs. membrane-bound Rab27a
Use of induced pluripotent stem cell models to probe the pathogenesis of choroideremia and to develop a potential treatment (17)	CHM REP1; JB-415, c.1327_1328delAT; JB-500, exon 2–4 deletion; JB-527, exon 2–4 deletion; JB-588, Arg555STOP, AGA→TGA	4	PBMCs	Non-integrating Sendai virus vector	2D, RPE	Healthy controls	Yes, AAV7m8	Mutant RPE: 1. Accumulation of unprenylated Rab protein 2. Failure of REP1 protein trafficking 3. Reduced phagocytic activity 4. Altered distribution of Rab27a

**TABLE 4:** iPSC-derived models of gyrate atrophy.

Publication	Gene; mutation	Number of patients	iPSC source	Re-programming method	Culture type	Control iPSC	Rescue	Relevant readouts
Optic vesicle-like structures derived from human pluripotent stem cells facilitate a customized approach to retinal disease treatment (47)	OAT; A226V	1	Fibroblast (Coriell)	Lentiviral transduction	3D, optic vesicle-like structures and RPE cells	Wild-type	Yes, BAC-mediated homologous recombination	Mutant RPE cells: 1. Reduced OAT activity 2. Greater enhancement of OAT activity when exposed to 600 μM vitamin B_6_ as compared to control

**TABLE 5:** iPSC-derived models of Stargardt disease.

Publication	Gene; mutation	Number of patients	iPSC source	Re-programming method	Culture type	Control iPSC	Rescue	Relevant readouts
Photoreceptor progenitor mRNA analysis reveals exon skipping resulting from the ABCA4 c.5461-10T→C mutation in Stargardt disease (99)	ATP binding cassette subfamily A member 4 (ABCA4); c.5461-10T>C	17 total, 3 iPSC lines	Fibroblast (skin biopsy)	Lentiviral transduction	2D, photoreceptor progenitors	Healthy controls	No	Mutant photoreceptor progenitor cells demonstrated exon-skipping splice defects in the mRNA
The intronic ABCA4 c.5461-10T > C variant, frequently seen in patients with Stargardt disease, causes splice defects and reduced ABCA4 protein level. (100)	ABCA4; c.5461-10T>C	4	Fibroblast (skin biopsy)	NA	2D, fibroblasts	Healthy controls	No	Mutant fibroblasts: 1. Exon-skipping splice defects in the mRNA 2. Reduced expression of full-length ABCA4 protein
Membrane attack complex mediates retinal pigment epithelium cell death in Stargardt macular degeneration (131)	ABCA4, c.3386G>T and c.5461-10T>C;5603A>T	1	Fibroblast (skin biopsy)	RNA reprogramming	2D, RPE	Healthy controls	No	Mutant RPE: 1. Reduced recycling of retinaldehyde 2. Increased MAC found on membranes
Cell-autonomous lipid-handling defects in Stargardt iPSC-derived retinal pigment epithelium cells (101)	ABCA4, c.6088C>T	1	Fibroblasts (skin biopsy)	Non-integrating Sendai virus vector	2D, RPE	Isogenic	No	Mutant RPE: 1. Increased intracellular lipid and apical ceramide accumulation during POS feeding 2. Reduced POS digestion

**TABLE 6:** iPSC-derived models of Best Vitelliform Macular Dystrophy.

Publication	Gene; mutation	Number of patients	iPSC source	Re-programming method	2D/3D	Differentiated cell type	Control iPSC	Rescue	Relevant readouts
iPS cell modeling of Best disease: insights into the pathophysiology of an inherited macular degeneration (105)	Bestrophin 1 (BEST1); A146K, N296K	2	Fibroblast (skin biopsy)	Lentiviral transduction	2D, RPE		2 sibling controls	No	Mutant RPE: 1. Disrupted fluid flux 2. Abnormal POS handling
Pharmacological modulation of photoreceptor outer segment degradation in a human iPS cell model of inherited macular degeneration (106)	BEST1; A146K, N296K	2	Fibroblast (skin biopsy)	Lentiviral transduction	2D, RPE		2 sibling controls	Yes	Mutant RPE demonstrated delayed POS degradation
Bestrophin 1 is indispensable for volume regulation in human retinal pigment epithelium cells (107)	BEST1; A243V, Q238R	2	Fibroblast (skin biopsy)	Lentiviral transduction	2D, RPE		Healthy control	No	Mutant RPE had reduction in volume-regulated anion channel-mediated currents
BESTROPHIN1 mutations cause defective chloride conductance in patient stem cell derived RPE (108)	BEST1; R218H, L234P, A243T	3	Fibroblast (skin biopsy)	Non-integrating Sendai virus vector	2D, RPE		Healthy control	No	Mutant RPE had defective Cl^–^ ion efflux
Patient-specific mutations impair BESTROPHIN1’s essential role in mediating Ca(2+)-dependent Cl(−) currents in human RPE (109)	BEST1; P274R, I201T	2	Fibroblast (skin biopsy)	Non-integrating Sendai virus vector	2D, RPE		Age-matched control	Yes, baculoviral vector	Mutant RPE had abolished Ca^2+^ dependent Cl^–^ current
Mutant Best1 expression and impaired phagocytosis in an iPSC model of autosomal recessive bestrophinopathy (110)	BEST1; R141H, I366fsX18	1	Fibroblast (skin biopsy)	Non-integrating Sendai virus vector	2D, RPE		Parental control	No	Mutant RPE: 1. Reduced BEST1 protein expression 2. Impaired phagocytosis

For studies that have conducted electrophysiological characterization, retinal organoids typically demonstrate light responses which are weaker than the mature mammalian retina (86). This is probably due to the lack of maturity of the underlying photoreceptor cells, evidenced by the shorter outer segment lengths. As the phototransduction cascade begins in the disc membrane of the outer segments, it is critical for this cellular component to reach maturity. Hence, the length of these outer segments can serve as an easily measured but indirect method of photoreceptor maturity. However, not all protocols have published the lengths of the produced outer segments. In the human retina, the RPE and photoreceptor work together as a functional unit with the RPE phagocytosing shed outer segments, recycling visual pigments, providing nutrients and growth factors. However, during 3D organoid differentiation, RPE cells typically form opposite the neural retina without close interaction. This has led to the hypothesis that the lack of RPE support is a contributing factor to immature photoreceptors. As differentiation protocols to date have not been able to encourage RPE – photoreceptor interaction, newer approaches have been developed to address this. Achberger et al. (115) developed a retina-on-a-chip platform by combining iPSC derived organoids and RPE in a microfluidic chip. This approached resulted in three times the number of outer segments developing in organoids compared to those grown without the RPE layer (115). While no electrophysiological characterization was demonstrated in this study, future improvements to this approach may potentially allow photoreceptors to mature, eventually achieving light responses that are similar to mature human photoreceptors.

### 5.2 Batch variability

The batch variability of both 2D and 3D differentiation protocols still needs to be significantly reduced. While methods for 2D RPE purification have achieved significant success, with some protocols achieving more than 90% functional RPE yields (116), 2D photoreceptor differentiation protocols still produce heterogenous cultures with the highest yield ranging around 60% (117). With the low yields of photoreceptors, the field slowly moved toward utilizing 3D retinal organoids as a source for photoreceptors for transplantation research. However, batch variability is also a significant hurdle for 3D retinal organoid protocols in order to ensure reproducibility of disease modeling readouts across organoids. In the production of 3D retinal organoids, iPSCs can also produce non-retinal cells instead. Hence, in multiple protocols, manual dissection of non-retinal tissue under light microscopy is recommended to ensure the heterogeneity of cultures (118).

A recent study by Tresenrider et al. (119) utilized single-cell sequencing to explore the phenomenon of retinal organoid batch variability. The study noted that organoids can be classified according to each organoid’s cell-class compositions. It was noted that with Wnt agonist treatment, more than 75% of the organoids were driven toward a single subtype defined by high retinal progenitor cell population and retinal cell counts. However, a secondary subtype still persisted. This subtype was defined by high RPE and roof plate cell content (119). Given that the molecular signature of this secondary subtype has been identified, future efforts can be channeled into developing automatic methods for identifying and sorting organoids based on this.

### 5.3 Capturing the anatomical architecture and cellular diversity of the human retina

The retina is a complex tissue that has a laminated structure with complex interplay between photoreceptor, RPE, ganglion, glial, and vascular cells. While 2D cultures do not demonstrate this laminated structure, 3D retinal organoids are better at replicating the retinal architecture, albeit still with some differences. The development of retinal organoids and appearance of cell types typically follows the order of retinogenesis. From retinal progenitor cells, retinal ganglion cells will first develop. This is followed by amacrine and horizontal cells before mature photoreceptors, bipolar and glial cells finally develop. Some key differences between retinal organoids and the human retina that still has to be improved includes the lack of spatial association of the RPE with the photoreceptors, the lack of vascularity and the declining number of retinal ganglion cells at the later stages of organoid development (118). In particular, as retinal ganglion cells are the primary output neurons of the retina, they are an important component of functional evaluation of the organoids.

Furthermore, to improve the ability of these 3D retinal organoids to capture the pathophysiology of diseases *in vitro*, the immune response and vascularity seen in native retina has to be replicated. For instance, with the ability to develop iPSC-derived choroidal endothelial cells (120), incorporation into 3D retinal organoids can allow the dissection of disease mechanisms in choroidal dystrophies where the disease development processes in the RPE/choroidal complex can be dissected. These diseases include central areolar, peripapillary, or diffuse choroidal dystrophy.

Another key challenge for 3D retinal organoids is the lack of the fovea. In human retina development, retinogenesis and maturation starts in the central retina prior to proceeding to the periphery. This results in the human retinal structure which has a central fovea centralis region with a dense cone population and perifoveal region containing a mix of cone and rod populations. Current retinal organoids protocols have been able to achieve cone rich neural retinal layers (121). While the cones have been investigated to have similar single-cell transcriptomes as the human macular cells, rod subtypes were still located throughout, making the recapitulation of the fovea centralis structure still challenging. Organ-on-a-chip approaches have been leveraged to capture the anatomical architecture and cellular diversity of the human retina. Maurissen et al. (122) developed an inner blood-retinal barrier-on-a-chip model by culturing primary human retinal microvascular cells into perfusable microvascular networks. The chip demonstrated both organization and expression of cellular markers that were similar to native tissue affected by diabetic retinopathy. The outer blood retinal barrier has also been modeled using organ-on-a-chip systems by (123). The team developed a chip that included a monolayer of human immortalized RPE and a microvessel of human endothelial cells, separated by a semi-permeable membrane. These models allow the study of various cellular interactions. Future work in retina-on-a-chip approaches can focus on understanding the crosstalk between photoreceptors, RPE and retinal ganglion cells. The ability to mimic retinal architecture also offers the opportunity to recapitulate the cellular structure and hopefully, the function seen in the human fovea.

Adaptive optics technology that is currently being used clinically provides the ability to image the human retina at the cellular level. The technology achieves this by correcting the optical aberrations inherent to the eye during imaging. The ability to evaluate the retina at a cellular level highlights new anatomical readouts that can be used to evaluate the ability of 2D and 3D cultures to mimic the retina. These parameters include cellular density, spacing, and regularity (124, 125). These insights can further guide the development of organoid or retina-on-a-chip technologies for more accurate biological representation.

### 5.4 Optimizing iPSC-derived retinal cellular models for drug discovery processes

Majority of drug candidates fail Phase III trials due to the lack of clinical efficacy. This has been purported to be due to the lack of biological relevant disease models in the pre-clinical development phase. The ability to utilize iPSC-derived retinal models for high-throughput drug discovery has the potential to improve the reliability of current drug discovery processes. While iPSC-derived retinal models will capture the underlying genetics associated with disease, the downstream biology including gene expression and cellular function still needs to be further studied. A key question for exploration is the difference between the iPSC-derived retinal models, animal models and diseased human tissue at the RNA transcriptome and proteome level. While large studies in age-related macular degeneration have suggested the adequacy of iPSC-derived cell lines in capturing the biology of human diseased tissue (126), there are currently no available studies for IRDs. The lack of available human samples due to the rarity of IRDs further impedes efforts in this area. Furthermore, there is a dearth of comparison of such datasets between iPSC-derived retinal models and in-vivo disease models. As the development of these iPSC-derived retinal cellular models progress to become assays with time, it is also important to compare the performance of such assays with current standards including assays utilizing human retinal tissue or commercial retinal cell lines.

## 6 Conclusion

Induced pluripotent stem cell-derived disease modeling technologies have advanced significantly in the retinal field. Despite the existing technological hurdles, various groups have already utilized these models to study novel therapeutic approaches such as gene therapy (127) and oligonucleotide therapeutics (80, 128, 129). However, future efforts at improving disease modeling efforts should focus on minimizing line-to-line variability, improving scalability of retinal organoid cultures, and ensuring appropriate controls to improve the reproducibility of experiments comparing the effects of various therapies.

The recent announcement of the FDA Modernization Act 2.0 heralds a unique time for drug development. With the act, drug developers can use novel human-relevant testing methods rather than traditional animal models for pre-clinical testing. Hence, the demand for relevant *in vitro* disease models will fuel the continued development of such technologies. With the incorporation of the abovementioned emerging technologies, human iPSC-derived IRD models can be expected to be a powerful tool in elucidating the pathogenesis of various IRDs.

## Author contributions

IS: Conceptualization, Methodology, Writing – original draft, Writing – review & editing. DG: Conceptualization, Methodology, Writing – original draft, Writing – review & editing. AB: Writing – original draft, Writing – review & editing. XS: Conceptualization, Funding acquisition, Methodology, Writing – review & editing.
